# Analysis of engineered T7 bacteriophages containing genetic sequences encoding antimicrobial peptides

**DOI:** 10.3389/frabi.2024.1515874

**Published:** 2025-01-15

**Authors:** Tobias Ludwig, Daniela Volke, Andor Krizsan

**Affiliations:** Institute of Bioanalytical Chemistry, Faculty of Chemistry and Mineralogy, Center for Biotechnology and Biomedicine, Leipzig University, Leipzig, Germany

**Keywords:** bacteriophages, antimicrobial peptides (AMP), melittin, CRAMP, apidaecins, synergy, phage engineering, OmpA signal peptide

## Abstract

Because of the global spread of multi- and pan-resistant bacteria, there is a need to identify, research, and develop new strategies to combat these pathogens. In a previous proof-of-concept study, we presented an innovative strategy by genetically modifying lytic T7 bacteriophages. We integrated DNA fragments encoding for derivatives of the antimicrobial peptide (AMP) apidaecin into the phage genome to induce the production and release of apidaecin within the T7 infection cycle, thereby also targeting phage-resistant *Escherichia coli* bacteria. In this follow-up study, we optimized the apidaecin encoding insert to improve the expression of the apidaecin derivative Api805 by adding the secretion signal peptide of the OmpA protein. This prevented the detrimental effects of the peptide on the producing bacterial cell after its production. The integration of two copies of the *OmpA*-Api805 insert into the phage genome resulted in T7Select-2x*OmpA*-Api805 phages, which had a partially improved activity in inhibiting phage-resistant *E. coli* compared to the T7Select phages without insert and with only one copy of the *OmpA*-Api805 insert. Additionally, we showed that the combinatorial use of the lytic bacteriophage T7Select with the highly active and lytic AMPs CRAMP (cathelicidin-related AMP) and melittin against *E. coli* made the lysis process of the phage and the peptides more effective and prevented the growth of potentially AMP- and phage-resistant *E. coli* strains. The integration of DNA sequences derived from CRAMP and melittin into the phage genome resulted in the created T7Select-(M)CRAMP and T7Select-(M)melittin phages, which showed a lysis behavior like the phage without insert and partially inhibited the growth of potentially phage-resistant *E. coli* strains after the phage-mediated lysis.

## 1 Introduction

The rise of antibiotic resistance has led to a global public health crisis (O’Neill, 2014). Bacterial infections that were once easily treated with antibiotics are now becoming increasingly difficult to cure due to the emergence of multidrug-resistant bacteria (Nikaido, 2009; van Duin and Paterson, 2016). In 2019, it was estimated that approximately 4.95 million deaths were associated with bacterial antimicrobial resistance, of which 1.27 million deaths were directly caused by the infection (Antimicrobial Resistance Collaborators, 2022). This death toll calls for the exploration of alternative strategies to combat bacterial infections, such as antimicrobial peptides (AMPs), bacteriophages, antisense therapeutics, immunotherapies, vaccines, probiotics, and microbiota-based therapeutics (Kumar et al., 2021; García-Contreras et al., 2022; MacNair et al., 2024).

The potential of AMPs has been recognized due to their broad-spectrum activity against various pathogens (Huan et al., 2020). Among the numerous AMPs identified, CRAMP (cathelicidin-related AMP), melittin, and apidaecin 1b have emerged as promising candidates due to their potent antimicrobial properties. CRAMP (**Table 1**), produced in mice, is an amphipathic, α-helical peptide that preferentially binds to negatively charged groups of bacterial membranes, thereby damaging them (Gallo et al., 1997). Melittin (**Table 1**), a component of bee venom, is a linear, water-soluble, cationic, amphipathic peptide (Memariani et al., 2020). It exerts its potent bactericidal activity by acting on the bacterial membrane, causing pore formation and disrupting membrane function, ultimately leading to lysis of the bacterial cells (Fidelio et al., 1984). Apidaecin 1b (GNNRPVYIPQPRPPHPRL), naturally produced by the honey bee (*Apis mellifera*), and its previously designed derivative Api805 (**Table 1**) belong to the group of proline-rich AMPs (PrAMPs), which are linear peptides with a high content of proline and arginine (Casteels et al., 1994; Li et al., 2006; Ludwig et al., 2022a). Apidaecins show remarkable efficacy against Gram-negative bacteria (Li et al., 2006), such as *Escherichia coli*, by entering the cell through the specific transporter SbmA and inhibiting the bacterial 70S ribosome (Krizsan et al., 2014, 2015; Ludwig et al., 2022a).

**Table 1:** Amino acid sequences of peptides synthesized* and/or expressed from coding DNA sequences^#^ in this study.

Peptide	Sequence	Molecular weight (g/mol)
CRAMP*	GLLRKGGEKIGEKLKKIGQKIKNFFQKLVPQPEQ	3,878.66
(M)CRAMP^#^	MGLLRKGGEKIGEKLKKIGQKIKNFFQKLVPQPEQ	4,009.86
Melittin*	GIGAVLKVLTTGLPALISWIKRKRQQ	2,847.49
(M)melittin^#^	MGIGAVLKVLTTGLPALISWIKRKRQQ	2,978.68
Api805*	GNNRPIYIPRPRPPHPRPIRV	2,502.96
Api805(G1M)*^/#^	MNNRPIYIPRPRPPHPRPIRV	2,577.10
*OmpA-*Api805^#^	MKKTAIAIAVALAGFATVAQAGNNRPIYIPRPRPPHPRPIRV	4,531.44
His_6_-LAPRGSV-Api805^#^	MHHHHHHLAPRGSVGNNRPIYIPRPRPPHPRPIRV	4,137.80

*OmpA*, signal peptide for periplasmic secretion of OmpA; His_6_, hexahistidine tag; LAPRGSV, Linker with cleavage sites for thrombin and elastase.

An alternative approach to targeting bacteria relies on bacteriophages, viruses that infect and destroy bacteria, which have gained increasing attention in recent years as potential therapeutics due to their remarkable specificity and ability to target bacterial strains including multidrug-resistant bacteria (Alqahtani, 2023; Kulshrestha et al., 2024). However, the efficacy of bacteriophages can be limited by several factors, including host-range limitations and the frequent emergence of phage-resistant bacterial strains (Zalewska-Piątek, 2023; Laanto, 2024). To overcome these challenges, synergistic interactions between bacteriophages and other antimicrobial agents have been investigated to enhance their bactericidal activity (Oechslin et al., 2017; Tagliaferri et al., 2019).

Previously, we successfully demonstrated that the combinatorial use of bacteriophage T7Select and apidaecins shows promising synergistic effects by inhibiting the regrowth of phage-resistant strains after phage lysis (Ludwig et al., 2022b). In a proof-of-concept study, we integrated DNA sequences encoding apidaecins into the T7Select phage genome downstream of the gp10 gene encoding the capsid protein to allow expression of apidaecins by the bacterial host (*E. coli*) during the phage infectious cycle (Ludwig et al., 2022b). With this approach, we aimed to improve the host range limitation of the phage by local production of apidaecin peptides, especially to combat phage-resistant bacterial subpopulations. However, the engineered phages did not show inhibition of phage-resistant bacterial regrowth or improved overall activity compared to the original T7Select phage because the expression levels of apidaecins were too low, even too low to be detected by mass spectrometry (Ludwig et al., 2022b).

**Figure 1:**
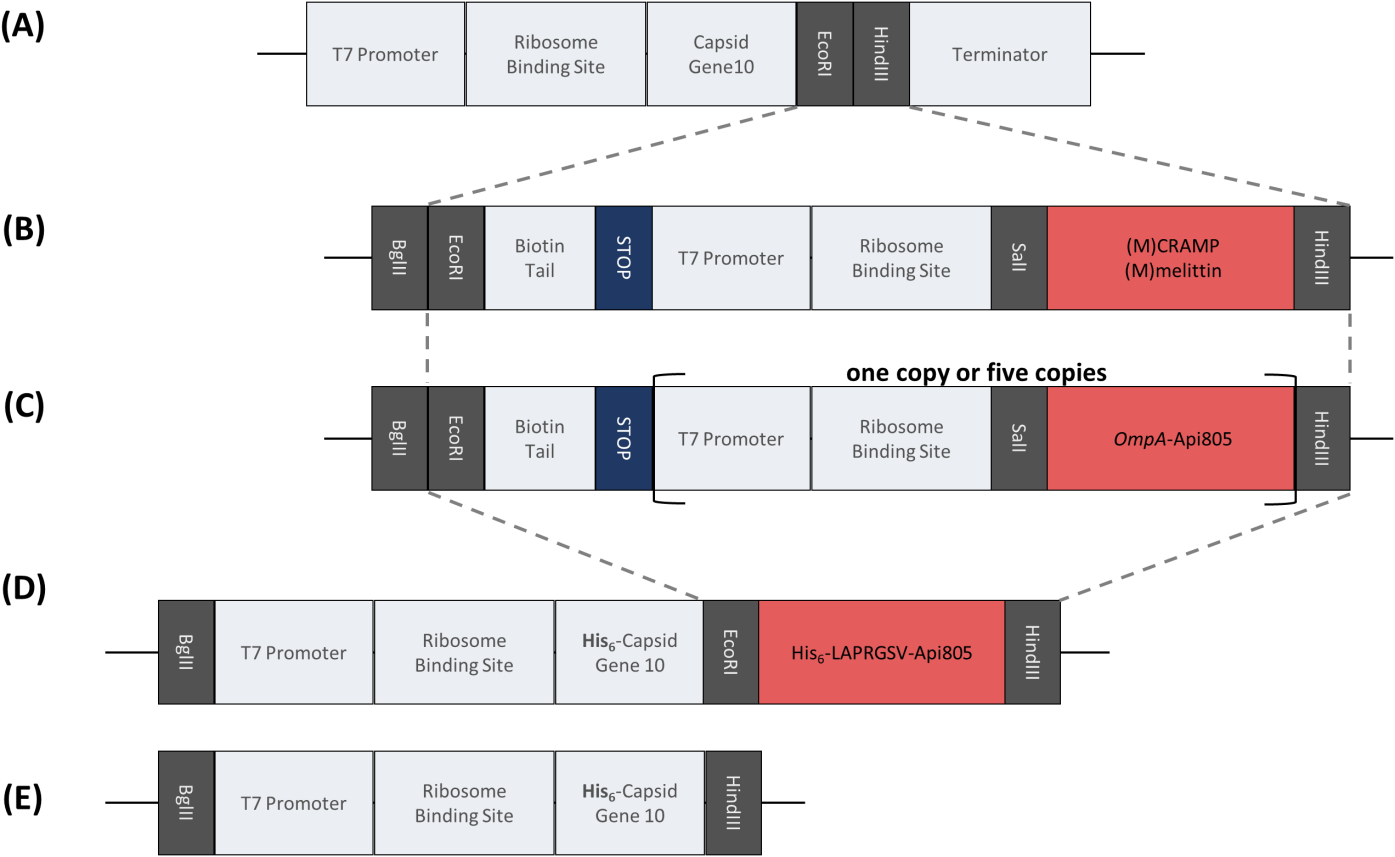
Overview of the peptide coding DNA fragments for integration into the pET28a(+) plasmid and the T7Select phage genome. Gray dashed lines indicate the part to be integrated into the phage genome. Schematic representation of the multiple cloning site in the T7Select phage genome **(A)**. Insert encoding the lytic AMPs (M)CRAMP and (M)melittin **(B)**. Insert encoding the fusion peptide of Api805 with N-terminal addition of the signal peptide of OmpA in one or five copies **(C)**. Insert encoding the direct attachment of Api805 to the viral capsid protein via a linker containing sequences for the hexahistidine tag and cleavage sites for thrombin and elastase **(D)**. Insert of capsid gene 10 for cloning into pET28a(+) for expression in *E*. *coli* **(E)**.

In this follow-up study, we report the synergistic effect of CRAMP and melittin with the T7Select phage against *E. coli* and the construction and characterization of T7Select bacteriophages (**Figures 1A, B**) engineered to express these membrane-active and lytic AMPs. These AMPs have a different mode of action than the ribosome-inhibiting apidaecins we used previously and therefore should not significantly interfere with their own expression at the 70S ribosome in the host cell. In addition, we aimed to enhance the expression of the apidaecin derivative Api805 by adding the secretion signal peptide of OmpA to the apidaecin sequence (**Figures 1A, C**), named *OmpA*-Api805 (**Table 1**), to prevent its intracellular accumulation with adverse effects on the producing host cell. This should improve peptide expression during bacterial growth and the phage infection cycle by secreting the peptides into the periplasm (Thie et al., 2008; Freudl, 2018; Erkut, 2021). Additionally, we aimed to increase the expression rate by inserting multiple copies of the *OmpA*-Api805 sequence into the phage genome (**Figures 1A, C**; **Supplementary Figure S1**). To avoid replication misalignments (“slippage”) and genetic rearrangements based on direct repeats of homologous DNA sequences (Bzymek and Lovett, 2001) in this case, we altered the codon usage for each *OmpA*-Api805 copy and intermediate sequences (**Supplementary Figure S1**). However, this was not possible for the repeated sequences of the T7 promoter and the ribosome-binding site within the insert. Another approach was to attach the Api805 peptide to the viral capsid via a cleavable linker (**Table 1**; **Figures 1A, D**).

## 2 Materials and methods

### 2.1 Materials and chemicals

Reagents used are listed in the **Supplementary File S1**. *E. coli* and phage strains, plasmids, and primers are listed in **Supplementary Table S1**. Amino acid sequences of peptides expressed from coding DNA sequences are listed in **Table 1**. Water was purified using a Purelab Ultra water purification system (electrical resistivity >18.2 kΩ×m; organic content <2 ppb; ELGA LabWater, Celle, Germany).

### 2.2 Peptide synthesis

Peptides were synthesized on a multiple synthesizer (SYRO2000, MultiSynTech GmbH, Witten, Germany) using the Fmoc/^t^Bu protection strategy, *in situ* activation with DIC in the presence of HOBt, and Wang resins to obtain the peptides as C-terminal acids. Peptides were cleaved with TFA containing 12.5% (v/v) of a scavenger mixture [ethanedithiol, m-cresol, thioanisole, and water; 1:2:2:2 (by vol)], precipitated with cold diethyl ether, and purified by RP-HPLC on a Jupiter C_18_-column (21.2 mm ID) using an aqueous acetonitrile gradient in the presence of 0.1% TFA. Peptide purities were determined by RP-HPLC on a Jupiter C_18_-column (4.6 or 2 mm ID). Monoisotopic masses were confirmed by matrix-assisted laser desorption/ionization time-of-flight mass spectrometry (MALDI-TOF-MS; 5800 Proteomic Analyzer; AB Sciex, Darmstadt, Germany).

### 2.3 Antibacterial activity

Minimum inhibitory concentrations (MICs) were determined at least twice in triplicate using a liquid broth microdilution assay in sterile 96-well plates with a total volume of 100 μL per well. Aqueous peptide solutions were serially diluted twofold in 50 µL of 25% MHBII or LB medium. Overnight cultures were diluted in 25% MHBII or LB medium to 1.5×10^7^ cells per mL and an aliquot of 50 μL was added to each well. The final cell count in each well was 7.5×10^6^ CFU/mL. The plates were incubated at 37°C for 20 ± 2 h. The turbidity of each well was measured at 600 nm. The MIC was defined as the lowest peptide concentration at which the turbidity did not exceed that of the medium alone.

### 2.4 Plaque assay

For the plaque assay, *E. coli* Rosetta pLysS was grown to 8×10^8^ CFU/mL in LB medium. Phage lysates were prepared in serial dilutions. An aliquot of the *E. coli* culture (250 µL) was transferred to molten LB overlay agar (4 mL, 0.75% agar), the diluted phage lysate (100 µL) was added, and the mixture was poured onto solid LB agar plates (1.5% agar). After the upper agar solidified, the plates were inverted and incubated overnight at 37°C. Plaques were counted and phage titers were calculated by multiplying the plaque counts by the dilution factor.

### 2.5 Phage-peptide synergy assay

*E. coli* Rosetta pLysS was grown in LB medium to a cell count of 9.2×10^8^ CFU/mL and transferred to a 96-well plate (130 µL/well). PrAMPs (10 µL) were added to a final concentration of 128 µg/mL and serially diluted down to 1 µg/mL. A phage solution (10 µL, 7.5×10^8^ PFU/mL) was added to achieve a final multiplicity of infection (MOI) of 0.0625 and a final volume of 150 µL per well. Optical density at 600 nm (OD_600_) was recorded in all wells every 15 min after 5 s of medium orbital shaking at a constant temperature of 37°C for a total of 30 h on a PARADIGM™ (Beckman Coulter, Salzburg, Austria).

### 2.6 DNA constructs for expression and genetic modification of phages

Construct designs of DNA sequences (**Figures 1B–E**) based on previously reported constructs (Ludwig et al., 2022b) were ordered from Genscript Biotech B.V. (Leiden, The Netherlands). These DNA fragments were cloned into pET28a+ plasmid using BgIII and HindII and transformed into chemically competent *E. coli* DH5α by heat shock. Plasmids were isolated and purified using the QIAprep^®^ Spin Miniprep Kit (Qiagen, Hilden, Germany). Insertion of the DNA sequences of interest was verified by PCR using the pET28 primer (**Supplementary Table S1**) and sequencing.

### 2.7 Peptide and protein expression

The pET28a+ plasmids with different inserts (**Supplementary Table S1**) were transformed into chemically competent *E. coli* Rosetta pLysS by heat shock. Bacterial cultures were incubated in LB medium (initial OD_600_ of 0.05) at 37°C and 200 rpm. When an OD_600_ of 0.3–0.4 was reached, the culture was split into two and IPTG (final concentration 1 mmol/L) was added to one of the two cultures. Both cultures were incubated at 37°C and 200 rpm for 3 h and the OD_600_ was recorded hourly. Bacterial cells were harvested (30 min, 4,000 *g*, 4°C) and analyzed for protein and peptide expression by SDS-PAGE with Coomassie staining. Cells were disrupted using a FastPrep-24™ 5G instrument (MP Biomedicals, Solon, OH, USA) and centrifuged (30 min, 21,000 *g*, 4°C). The supernatant (soluble protein fraction) and the pellet resuspended in PBS containing urea (4 mol/L, insoluble protein fraction) were analyzed by SDS-PAGE.

### 2.8 Genetic modification of phages

Genetically modified phages were generated using the T7Select 415 Cloning Kit (Merck Millipore, Burlington, USA) according to the manufacturer’s instructions. The DNA sequences to be inserted (**Figures 1B–D**) were amplified by PCR using pET28a+ plasmids and pET primers (**Supplementary Table S1**). The PCR product was digested with the restriction enzymes EcoRI and HindIII (both New England Biolabs, Ipswich, USA) and then ligated into the predigested T7Select vector arms using T4 ligase (New England Biolabs, Ipswich, USA) at 4°C for 16 h. The ligated T7Select genome was added to the provided packaging extract. Packaging was performed for 2 h at room temperature, followed by a plaque assay to obtain infectious particles. Formed plaques were transferred to LB medium (450 µL), incubated at 37°C for 2 h, and chloroform was added (33 µL). For phage propagation and larger lysate production, *E. coli* Rosetta pLys was grown in LB medium at 37°C to an OD_600_ ≈ 0.5–1.0 and then 100 µL of undiluted phage lysate was added to 5 mL of bacterial culture. The mixture was then incubated at 37°C until complete lysis, the mixtures were centrifuged (5 min, 5,000 *g*), sterile filtered (22 μm) in glass culture tubes, and stored at 4°C. Proper insertion was verified by PCR using T7 primers (**Supplementary Table S1**) and sequencing.

### 2.9 Time-kill assay

For the time-kill assay, *E. coli* Rosetta pLysS was grown to 9.2×10^8^ CFU/mL in LB medium and transferred to a clear 96-well plate (130 µL/well). Phage lysate (20 µL, 3.75×10^8^ PFU/mL) was added to achieve a MOI of 0.0625. The outer edge of the 96-well plate, i.e., rows A and H and columns 1 and 12, was filled with 150 µL of sterile medium to reduce edge effects. The plate was incubated at 37°C for at least 30 h and OD_600_ was measured every 15 min after orbital shaking at medium speed for 5 to 10 s.

### 2.10 Mass spectrometry

One hundred microliters of bacteria cell culture supernatant, bacteria lysate, or phage lysate were mixed with 1.9 mL of 5% (v/v) acetonitrile in 50 mmol/L ammonium bicarbonate (pH 8.5, ABC solution). A 10-mg weak cation exchange cartridge (Oasis WCX, Waters Corporation, Milford, Massachusetts, USA) was wetted with 1 mL of acetonitrile and equilibrated with 1 mL of ABC solution. Samples were applied in two steps each 1 mL. To wash out unbound material, the cartridge was washed with 1 mL of ABC solution and 1 mL of 5% (v/v) acetonitrile in water with 2% (v/v) formic acid. Peptides were eluted with two steps each 0.3 mL of 60% (v/v) acetonitrile in water with 2% (v/v) formic acid to a low-bind 1.5-mL Eppendorf tube. Forty microliters of the eluate was mixed with 40 µL of 2% (v/v) formic acid in water and transferred to a polypropylene insert (Agilent Technologies, USA). WCX cleaned samples were measured with an M-class UPLC system (Waters, Manchester, UK) coupled online to an ESI-IMS-QTOF instrument (Synapt G2-Si, Waters, Manchester, UK). Sample (5 µL) was injected to Acquity BEH300 C4 trap column (1.7 µm, 180 µm × 20 mm, Waters, Ireland). Trapping was done at a flow rate of 5 µL/min at 20% B, where B was acetonitrile with 0.1% (v/v) formic acid and A was water with 0.1% (v/v) formic acid, for 6 min. The trapped sample was eluted to a BEH300 C_4_ column (1.7 µm, 100 µm × 100 mm, Waters, Ireland) and equilibrated at 20% B at a flow rate of 1 µL/min with a linear gradient from 20% B to 90% B in 10 min. Re-equilibration before next sample injection was done by 5 min back to 20% B and 14 more minutes at 20% B. Sample ionization was done with a Nanolockspray source (Waters, Manchester, UK) set up with a pre-cut PicoTip^®^ emitter (360 µm OD × 20 µm ID, 10 µm tip 2.5″ length, Waters, Milford, USA) at 3 kV. During the whole measurement every 45 s, the signal of a 100 fmol/µL GluFib solution (*m/z* 785.843 doubly charged ion signal) was measured for 0.5 s for mass correction. The following were the nano source parameters: 30 V sample cone, 80 V source offset, 100°C source temperature, 20 L/h cone gas, 0.2 bar nano flow gas, and 150 L/h purge gas, with 2 mL/min trap gas (nitrogen, argon for trap, and transfer collision cell), 180 mL/min helium for helium cell, and 90 mL/min IMS cell gas (nitrogen). TriWaveDC settings were 1 V for Trap DC entrance, 2 V for Trap DC bias, −2 V Trap DC, and 0 V Trap DC exit. TriWave IMS settings were 1,000 m/s wave velocity and 40 V wave height. Mass spectra were recorded with MassLynx 4.2 SCN 983 with the following HD-MRM method. Positive polarity was determined with an analyzer in resolution mode from 0 to 30 min for *m/z* range 50 to 2,000 with a scan rate of 0.5 s. Every 5 s, a radar scan was recorded. Using MRM scan padding, MS/MS was triggered from 10 to 20 min for *m/z* 570.10, 599.76, 600.40, 606.10, 634.36, 776.30, and 805.67 using transfer collision cell from 18 to 25 V for fragmentation. For data analysis, MassLynx 4.2 SCN983 and Drift Scope 2.9 (Waters, Manchester, UK) were used.

## 3 Results

### 3.1 Activity of AMPs and AMP-phage synergy against *E. coli*

The recently reported apidaecin derivatives Api805 (Ludwig et al., 2022a) and Api805(G1M), also named Api806 (Ludwig et al., 2022b), showed MIC values against *E. coli* Rosetta DE3 pLysS of 4 and 8 µg/mL in 25% MHBII, but only 128 µg/mL in LB medium (**Table 2**). A similar medium dependence was observed for the activity of CRAMP and melittin with MICs of 16 µg/mL in 25% MHBII and MICs of 128 and 32 µg/mL in LB medium, respectively. Similar activities were observed for CRAMP, Api805, and Api805(G1M) against the previously isolated T7 phage-resistant *E. coli* Rosetta DE3 pLysS strain R 2.3 (Ludwig et al., 2022b), whereas melittin was four- and eightfold more active in 25% MHBII and LB, respectively, with MICs of 4 µg/mL in both media (**Table 2**).

**Table 2:** MICs of antimicrobial peptides against wild-type *E. coli* Rosetta DE3 pLysS and the T7Select resistant strain R 2.3 (7.5×10^6^ CFU/mL) cultured in 25% MHB and LB media.

*E. coli* Rosetta DE3 pLysS strain	MIC (µg/mL)							
	CRAMP		Melittin		Api805		Api805(G1M)	
	25% MHBII	LB	25% MHBII	LB	25% MHBII	LB	25% MHBII	LB
Wild type	16	128	16	32	4	128	8	128
R2.3	16	128	4	4	4	128	4	128

**Figure 2:**
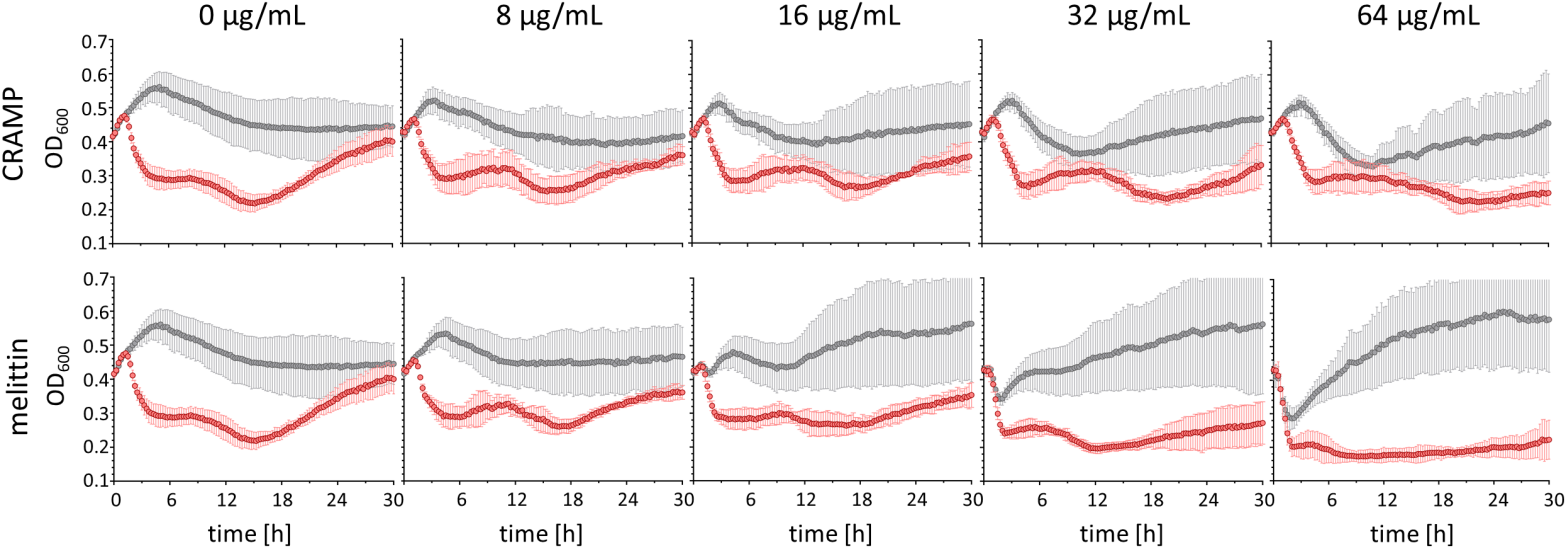
Bacterial growth rates of *E. coli* Rosetta (*t*_0_: ~1.2×10^8^ CFU) uninfected (gray) and infected (red) with Phage T7Select (MOI = 0.0625), and increasing concentrations of the lytic AMPs CRAMP and melittin. Shown are OD_600_ values recorded every 15 min after 5 s shaking for 30 h. Experiments were performed twice as single determinations and once as duplicates. Error bars indicate the standard deviation of all four replicates.

Since ribosome-targeting apidaecin derivatives have been reported to act synergistically with the T7Select phage in a certain concentration range without interfering with phage-mediated lysis, but preventing or delaying regrowth of phage-resistant bacteria (Ludwig et al., 2022b), the effect of the lytic AMPs CRAMP and melittin added to an uninfected and T7Select phage-infected *E. coli* Rosetta culture was investigated (**Figure 2**). Uninfected *E. coli* Rosetta pLys grew in LB medium from an OD_600_ of ~0.4 (~1.2×10^8^ CFU) measured in a 96-well plate to an OD_600_ of ~0.55 within 5 h before the OD_600_ decreased steadily over the next 13 h to ~0.45, which remained stable for the next 12 h. The addition of CRAMP and melittin resulted in different lysis patterns and different regrowth effects after initial lysis, which started at a concentration of 16 µg/mL and further increased with higher AMP concentrations (**Figure 2**). When incubated with 64 µg/mL CRAMP, the bacteria grew to an OD_600_ of ~0.5 in the first 3.5 h. The OD_600_ then decreased steadily over the next 7 h to ~0.35 before increasing to ~0.45 after 30 h of cultivation (**Figure 2**). Melittin added at the same concentration immediately lysed the bacteria, as indicated by a decrease in OD_600_ from ~0.4 to ~0.3 in the first 2 h. In the following 20 h, a strong regrowth to an OD_600_ of ~0.6 was observed, which remained stable for the rest of the cultivation period (**Figure 2**).

In the phage-infected *E. coli* Rosetta pLys cultures, lysis was triggered 1.5 h after phage addition in the absence of AMPs, reducing the OD_600_ from ~0.5 to ~0.3 in 3 h and further to ~0.2 in the following 10 h. However, regrowth started after approximately 18 h with a steady increase in OD_600_ to ~0.4 after 30 h of cultivation (**Figure 2**). The addition of CRAMP did not significantly affect the total lysis phase in the concentration range tested up to 64 µg/mL, but slightly reduced regrowth at 32 µg/mL and completely prevented it at 64 µg/mL over the entire observation period (**Figure 2**). Melittin addition to a phage-infected *E. coli* culture induced lysis earlier in a concentration-dependent manner starting at 16 µg/mL and at concentrations of 32 and 64 µg/mL, and the lysis was already completed after 90 min, presumably due to the strong lytic activity of melittin. In addition to a faster lysis, the lysis at 64 µg/mL also appeared to be more effective. As with CRAMP, melittin reduced regrowth at 32 µg/mL and completely inhibited it at 64 µg/mL (**Figure 2**). It should also be noted that the regrowth induced by the peptides was prevented by the addition of the phage. Thus, CRAMP and melittin appear to be promising candidates for combinatorial use with phages and also for phage engineering, since the lytic activities of phage and peptides were not reduced, but enhanced, and the regrowth of potentially resistant bacteria observed with phage and peptide treatments alone was inhibited.

### 3.2 Growth effects induced by the expression of apidaecin constructs and lytic AMPs in *E. coli*

**Figure 3:**
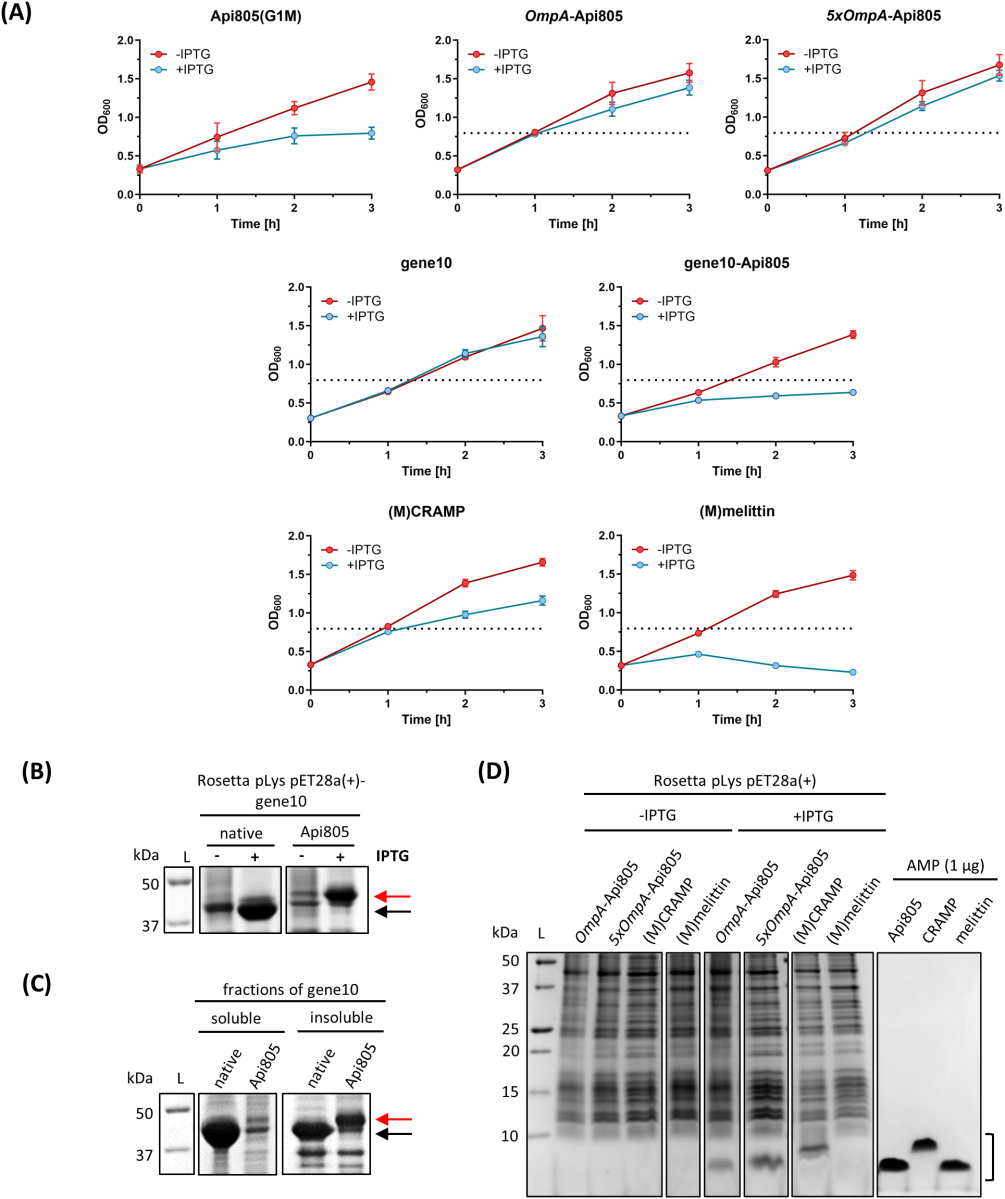
Bacterial growth rates of *E*. *coli* Rosetta harboring pET28a(+) vectors containing sequences for AMPs and for gene10 constructs. Growth rates of *E*. *coli* cultures were monitored by OD_600_ values without (red) or with (blue) IPTG-induced peptide/protein expression **(A)**. The horizontal black dotted lines show the growth maximum of Rosetta pLys pET28a(+)-Api805(G1M) (OD_600_ = 0.795) in the presence of IPTG for comparison. Experiments were performed twice in duplicate. Error bars indicate the standard deviation of all four replicates. Protein/peptide expression in *E*. *coli* Rosetta harboring pET28a(+) vectors containing sequences for gene10 constructs and AMPs **(B–D)**. SDS-PAGE (12%) with Coomassie staining of the corresponding *E*. *coli* preparations after 3 h of IPTG induction **(B)** and of the soluble and insoluble fractions after bacterial cell disruption **(C)**. The arrows indicate where the T7 capsid protein gene10 (black) and gene10-Api805 (red) migrate in the gel. SDS-PAGE (16% gel) with Coomassie staining of samples prepared from *E*. *coli* cells expressing peptides after 3 h of IPTG induction and of synthetic AMPs (1 µg) for comparison **(D)**. The black bracket indicates the region where the AMPs migrate in the gel.

Next, the ability of *E. coli* to express CRAMP, melittin, Api805, *OmpA*-Api805, and the fusion protein gene10B-Api805 was tested. Plasmids encoding Api805(G1M), *OmpA*-Api805, 5x*OmpA*-Api805, gene10-Api805, (M)CRAMP, and (M)melittin were transformed into *E. coli* Rosetta pLysS. Bacterial growth was monitored with and without inducing expression from the plasmid using IPTG, and their protein patterns were analyzed by SDS-PAGE (**Figure 3**). Induced expression of Api805(G1M) significantly reduced bacterial growth compared to the uninduced control with a difference in OD_600_ values of 0.662 ± 0.017, similar to gene10-Api805 (ΔOD_600_ = 0.758 ± 0.037), whereas expression of native T7 capsid protein (gene10), *OmpA*-Api805, and 5x*OmpA*-Api805 did not reduce bacterial growth (**Figure 3A**). Induction of (M)CRAMP expression reduced bacterial growth less than Api805(G1M), but still with an ΔOD_600_ of 0.496 ± 0.076 (**Figure 3A**). The strongest effect was observed for (M)melittin, which strongly reduced bacterial growth even below the baseline value, indicating a bactericidal effect due to cell lysis upon (M)melittin expression.

Both gene10 proteins displayed bands on SDS-PAGE, with the gene10-Api805 band being weaker than the native capsid protein gene10 band (**Figure 3B**). In contrast to the native capsid, the fusion protein gene10-Api805 was mostly present in insoluble inclusion bodies (**Figure 3C**). Similar to the previously reported Api805(G1M), also named Api806 (Ludwig et al., 2022b), the Api805 derivatives *OmpA*-Api805 and 5x*OmpA*-Api805 were also detected in the cell pellet after 3 h of induction as bands with apparent molecular weights below 10 kDa in SDS-PAGE (**Figure 3D**). However, the peptides were not detected at the expected molecular weight of ~4.5 kDa, but at an apparent weight of ~2.5 kDa, corresponding to Api805 (**Figure 3D**; **Table 1**). Since only Api805 without the OmpA signal peptide and no growth reduction for Rosetta pLys pET28a(+)-*OmpA*-Api805 and pET28a(+)-5x*OmpA*-Api805 (**Figure 3A**) were detectable, we assumed the expression of *OmpA*-Api805, followed by the natural cleavage of the signal peptide and the secretion of Api805 into the periplasm away from the immediate vicinity of the 70S ribosome, as originally designed. In contrast to (M)CRAMP, no visible band was detectable for (M)melittin in the cell pellet (**Figure 3D**). Analyzed by mass spectrometry after a WCX-SPE, it was possible to detect (M)melittin mainly in the bacteria culture supernatant and also in the bacterial lysate after lysing the residual cell pellet (**Supplementary Figure S2**). However, the peptide was not detected in its native variant, but with different modifications. (M)melittin from bacterial lysate was modified at the methionine in position 1 by oxidation (+15.999 Da, **Supplementary Figure S2A**). (M)melittin identified in bacterial culture supernatant was modified at the methionine like in the lysate (+15.999 Da on methionine); additionally, the tryptophane in position 20 was modified to kynurein (+3.989 Da) and N-formylkynurein (+31.999 Da, **Supplementary Figure S2B**).

### 3.3 Generation and activity of AMP-phages

**Figure 4:**
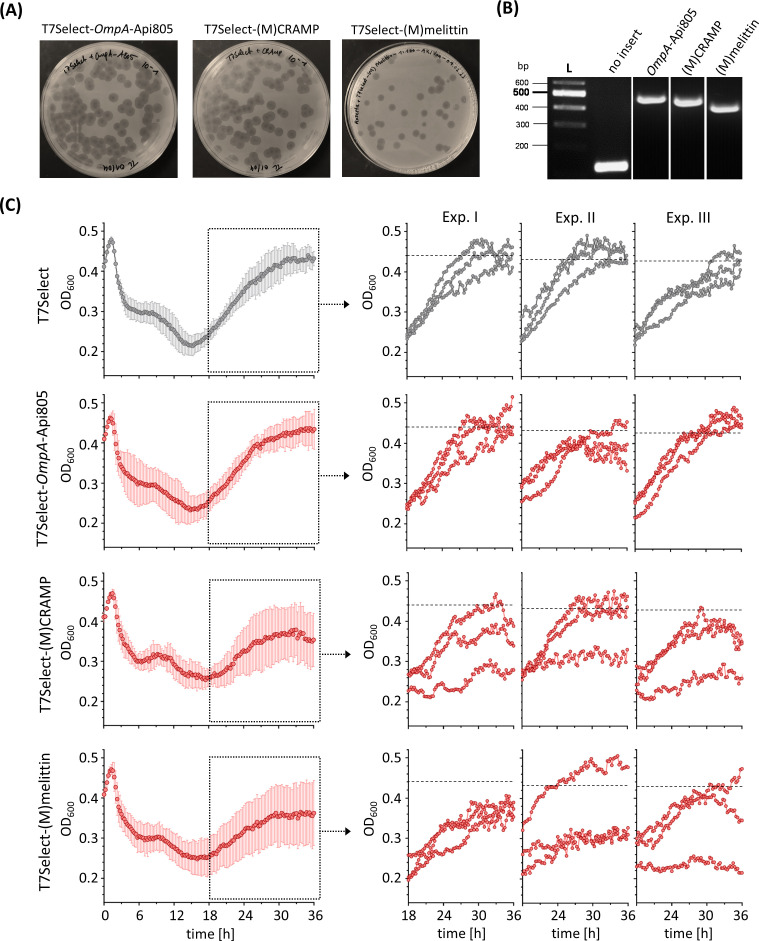
A plaque assay of engineered T7Select phages encoding different AMPs showing the recovery of infectious phages **(A)**. Ethidium bromide-stained agarose gel electrophoresis of DNA fragments amplified by PCR from phage genomes of engineered T7Select phages with T7 fwd and T7 rev primers to confirm the insertion of the AMP inserts *OmpA*-Api805 (483 bp), (M)CRAMP (462 bp), and (M)melittin (438 bp) **(B)**. Bacterial growth of *E*. *coli* Rosetta cultures infected with engineered T7Select phages (MOI 0.0625). Infected cultures with T7Select phage without insert (gray) and with inserts (red) encoding for *OmpA*-Api805, (M)CRAMP, and (M)melittin **(C)**. Shown are the OD_600_ values recorded every 15 min after 5 s shaking for 36 h Experiments were performed three times in triplicate. Shown are the curves with error bars (standard deviation) of all nine replicates over the entire cultivation period and the curves of the three individual replicates of each experiment (Exp.) in the regrowth phase, the cultivation period from 18 to 36 h. The horizontal black dotted lines show the mean growth of the culture infected with T7Select after 36 h of the respective experiment for comparison (Exp. 1: OD_600_ = 0.439 ± 0.021/Exp. 2: OD_600_ = 0.430 ± 0.008/Exp. 3: OD_600_ = 0.426 ± 0.017).

T7Select phage constructs encoding *OmpA*-Api805, (M)CRAMP, and (M)melittin sequences (**Figures 1A–C**) resulted in infectious particles (**Figure 4A**). Analysis of the inserted DNA fragments of the engineered phage genomes by PCR and agarose gel electrophoresis compared to the unmodified T7Select phage showed the correct size confirming the insertion of *OmpA*-Api805, (M)CRAMP, and (M)melittin (**Figure 4B**). The phages were genetically stable with respect to the insert, which could be detected and confirmed in all plaques tested even after repeated propagations and in produced phage lysates. Phage lysates of T7Select-*OmpA*-Api805, T7Select-(M)CRAMP, and T7Select-(M)melittin prepared immediately after phage-mediated lysis were analyzed for AMP production by mass spectrometry after a WCX-SPE. (M)melittin expression was confirmed (**Supplementary Figure S4**), but as with the bacterial expression samples (**Supplementary Figure S2**), the peptide was not detected in its native variant, but was modified with a mass shift of +44.990 Da at the methionine (**Supplementary Figure S3**), which could be due to a carboxylation. However (M)CRAMP, *OmpA*-Api805, or free Api805 could not be detected by the mass spectrometry method applied. Most likely, (M)CRAMP and Api805 could not be detected due to low amounts, which could be attributed to inefficient expression or loss of the peptides owing to binding to various lysate or medium components during sample preparation. In addition, as with melittin, special modifications could shift the mass of the peptides to quite different *m/z* values, making it difficult to distinguish between the peptides and all the other components in the phage lysate.

The activity of the engineered phages T7Select-*OmpA*-Api805, T7Select-(M)CRAMP, and T7Select-(M)melittin was determined by a time-kill assay (**Figure 4C**; **Supplementary Figure S4**), performed three times in triplicates. The nine replicates of *E. coli* cultures infected with the control T7Select phage were very uniform and showed no significant variation in growth during the initial lysis and regrowth phases (cultivation phase from 18 to 36 h). The T7Select-*OmpA*-Api805 phage behaved like the control. The phages with the inserts for (M)CRAMP and (M)melittin also showed similar lysis behavior, but in contrast to the control, the *E. coli* cultures showed different growth behavior in the regrowth phase (**Figure 4C**; **Supplementary Figure S4**). Some replicates behaved similarly to the control, while others showed reduced or even inhibited regrowth, which indicate the presence of phage-induced AMPs.

**Figure 5:**
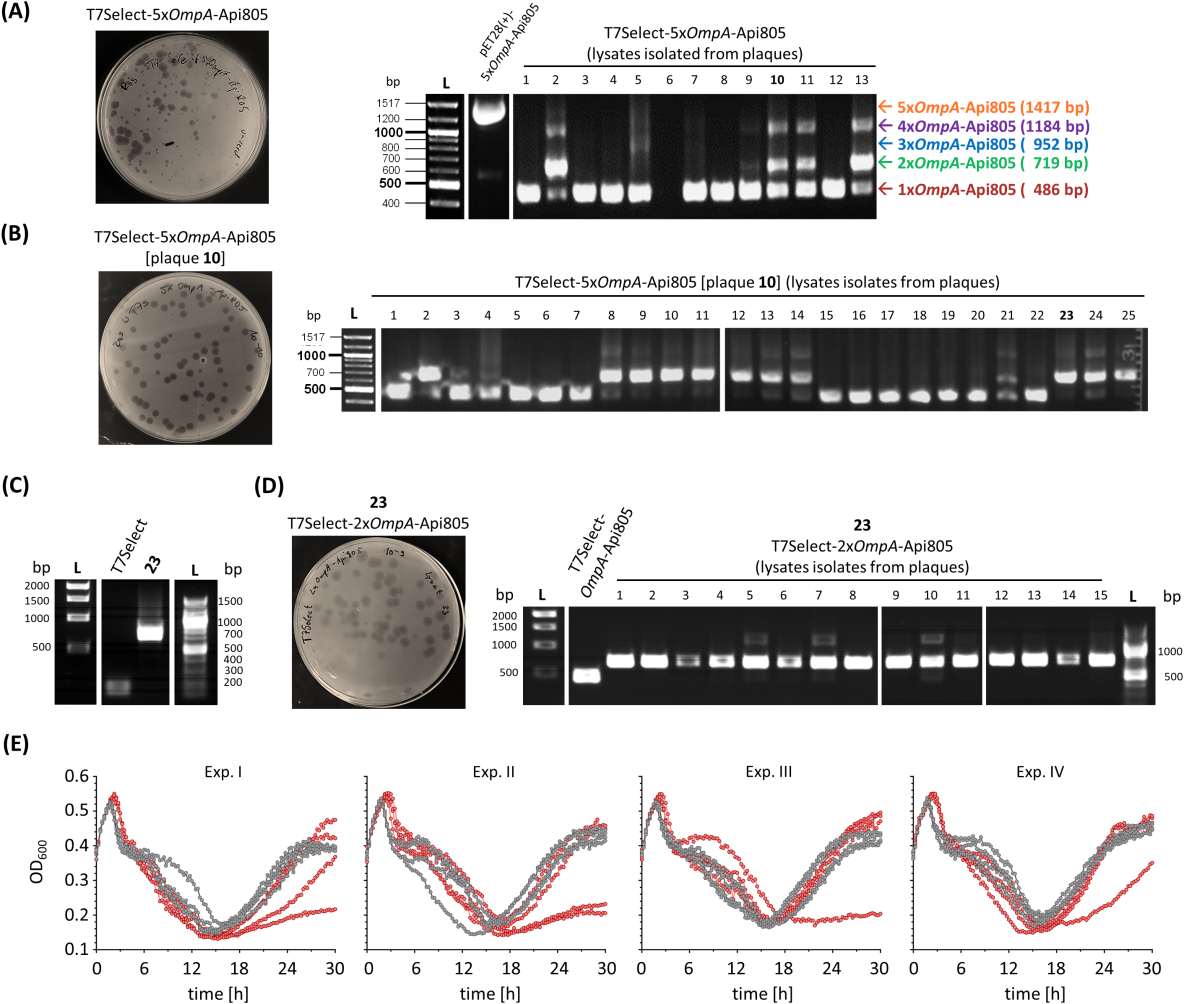
Agar plate after a plaque assay using the generated T7Select-5x*OmpA*-Api805 phage lysate against *E*. *coli* Rosetta and ethidium bromide-stained agarose gels of PCR-amplified DNA fragments from pET28(+)-5x*OmpA*-Api805 and T7Select-5x*OmpA*-Api805 phage lysates isolated from 13 different plaques **(A)**. Agar plate after a plaque assay using the T7Select-5x*OmpA*-Api805 phage lysate isolated from plaque 10 against *E*. *coli* Rosetta and ethidium bromide-stained agarose gel of DNA fragments from T7Select-5x*OmpA*-Api805 [plaque 10] phage lysates isolated from 25 different plaques **(B)**. Ethidium bromide-stained agarose gel of PCR-amplified DNA fragments from T7Select lysate and lysate 23 after an up-scaling propagation, which was confirmed as T7Select-2x*OmpA*-Api805 phage lysate **(C)**. Agar plate after a plaque assay using the T7Select-2x*OmpA*-Api805 phage lysate against *E*. *coli* Rosetta and ethidium bromide-stained agarose gel of DNA fragments from T7Select-2x*OmpA*-Api805 phage lysates isolated from 15 different plaques **(D)**. Bacterial growth of *E*. *coli* Rosetta cultures infected with T7Select (gray) and T7Select-2x*OmpA*-Api805 (red) with a MOI of 0.0625 **(E)**. Shown are the OD_600_ values recorded every 15 min after 10 s shaking for 30 h Experiments were performed four times in quadruplicates. Shown are the curves of the four individual replicates for each phage of each experiment (Exp.) over the entire cultivation.

Integration of the 5x*OmpA*-Api805 construct (**Figures 1A, C**) into the phage genome also resulted in infectious particles (**Figure 5A**). Thirteen plaques of different sizes were picked and the subsequently prepared phage lysates were tested for the insert by PCR and agarose gel electrophoresis (**Figure 5A**). All lysates except lysate 6 contained phages with a single copy of *OmpA*-Api805; only lysates 2, 10, 11, and 13 also contained phages with two and four copies of *OmpA*-Api805. Notably, no lysate was found to contain phages with the full insert of five copies of *OmpA*-Api805 (**Figure 5A**). No correlation between plaque size and copy number was observed. Lysate 10 was selected for a subsequent plaque assay (**Figure 5B**) that attempted to separate phages with different copy numbers of *OmpA*-Api805. Lysates of 25 plaques were analyzed. While 13 lysates contained mainly phages with only one copy of *OmpA*-Api805, 11 lysates mainly contained phages with two copies (**Figure 5B**). One lysate (21) showed faint bands of DNA fragments for one, two, and four copies. Lysate 23 was selected for a subsequent up-scaling propagation and a plaque assay. The phages from lysate 23 were confirmed to be T7Select-2x*OmpA*-Api805 carrying a genetically stable insert with two copies of *OmpA*-Api805 (**Figures 5C, D**), indicating that multiple copies can be incorporated into the phage genome by the chosen strategy. Sequencing of the T7Select-2x*OmpA*-Api805 genome confirmed the insertion of two copies of *OmpA*-Api805. Comparison with the 5x*OmpA*-Api805 sequence revealed a genetic rearrangement of the insert, with the first and fifth copy of the original 5x*OmpA*-Api805 insert inserted, whereas copies two, three, and four were deleted (**Supplementary Figures S1**, **S5**). Compared to the control, the T7Select-2x*OmpA*-Api805 phage with two copies of *OmpA*-Api805 showed no differences in the lysis behavior in a time-kill assay. Interestingly, 4 *E. coli* culture replicates showed reduced and 2 showed delayed regrowth of potentially phage-resistant populations, while the 10 others regrew similarly to the control (**Figure 5E**). This was overall an improvement over the single-copy *OmpA*-Api805 phage.

Cloning of sequences encoding His_6_-LAPRGSV-Api805 without an additional stop codon, T7 promoter, and ribosome binding site (**Figures 1A, D**), which should result in direct attachment of the peptide to the capsid, did not yield infectious phages.

## 4 Discussion

Phages are considered a potential alternative therapeutic strategy to overcome disadvantages of conventional antibiotics. However, their therapeutic applications are limited by significant pharmacological drawbacks. One major limitation is that most phages have a narrow host range targeting only specific bacterial strains (Sulakvelidze et al., 2001; Du et al., 2023). While this is not necessarily a drawback, it hinders their use as broad-spectrum antimicrobial agents. Another challenge is that even phage-susceptible bacteria can rapidly develop resistance, sometimes during the first treatment (Oechslin et al., 2017; Zalewska-Piątek, 2023). Our results consistently confirmed the emergence and proliferation of phage-resistant bacteria. Extending recent research showing a cooperative effect between phages and standard antibiotics or apidaecin-derived PrAMPs (Tagliaferri et al., 2019; Ludwig et al., 2022b), we also observed such dose-dependent synergistic effects for membranolytic CRAMP and melittin in combination with T7Select phages. The lytic activity of either phage or peptide was independent of each other and was not reduced when combined; in some cases, the effects were even enhanced. Regrowth of bacteria resistant or less susceptible to phages or peptides was inhibited when both antibacterial agents were combined. The significantly improved activity of melittin against the T7Select-resistant *E. coli* strain R 2.3 confirmed an indirect synergistic effect, because the adaptation of the bacterial host to the phage increases the sensitivity to the AMP. Considering previous studies by Qimron et al., Mutalik et al., and Ebbensgaard et al., it is most likely that gene deletions affecting the composition and type of lipopolysaccharide (LPS) structure in the outer membrane trigger both phage resistance and increased susceptibility to AMPs, including melittin (Qimron et al., 2006; Ebbensgaard et al., 2018; Mutalik et al., 2020).

Based on our results, we have developed genetically engineered lytic phages to specifically target phage-susceptible bacteria by inducing CRAMP and melittin expression in the host, which could attack nearby phage-resistant bacteria upon release from the lysed host cell. The partial reduction and inhibition of regrowth by these engineered phages provided significant improvements over the original T7Select phage and phages harboring apidaecin-derived sequences (Ludwig et al., 2022b). Engineering of the phage genome by insertion of sequences encoding CRAMP and melittin was demonstrated and the desired AMP production after phage infection and host lysis could be confirmed for (M)melittin, but not for (M)CRAMP, which does allow, at least for (M)melittin, a direct link between regrowth inhibition and AMP production. Although these effects were not fully reproducible, most likely due to different expression levels of (M)melittin and also (M)CRAMP in the single replicates, they provide a solid base for further development.

The T7Select 415-1b phage display kit and alternative methods have been successfully used to integrate sequences encoding peptides or proteins (Chen et al., 2017; Lemon et al., 2019; Ludwig et al., 2022b; Du et al., 2023; Meile et al., 2023) including antimicrobial agents, such as the anti-biofilm protein dispersin B (Lu and Collins, 2007), biofilm disrupting AMP 1018 (Lemon et al., 2019), the leaderless bacteriocin lacticin Q (Masuda et al., 2021), colicin-like bacteriocins (Du et al., 2023), and apidaecins (Ludwig et al., 2022b). However, phage-mediated expression was only confirmed for superfolder green fluorescent protein (sfGFP), fluorescent protein mCherry, *β-*galactosidase, and luciferase (Chen et al., 2017; Lemon et al., 2019; Ludwig et al., 2022b; Meile et al., 2023), whereas antimicrobial agents could not be detected after phage lysis, even by sensitive mass spectrometry methods (Lu and Collins, 2007; Lemon et al., 2019; Masuda et al., 2021; Ludwig et al., 2022b), despite improved activities of the engineered phages (Lu and Collins, 2007; Lemon et al., 2019; Masuda et al., 2021; Du et al., 2023). Therefore, based on the current data and to the best of our knowledge, the detection of (M)melittin is the first direct evidence of phage-induced AMP expression.

In contrast to the expression of Api805(G1M), the N-terminal extension of Api805 with the OmpA signal peptide reversed the reduction in bacterial growth rate upon IPTG-induced expression. This was expected since the addition of the OmpA signal peptide should trigger peptide translocation into the periplasm and thus reduce its intracellular concentration and inhibitory effect on the bacterial ribosome. When integrated into the phage genome, this modification had no impact on phage activity. It was also possible to introduce multiple sequences of *OmpA*-Api805 into the phage genome, although this was genetically instable, ultimately leading to phage genomes carrying two or one encoded *OmpA*-Api805 sequence. In contrast to the intermediate sequences and the *OmpA*-Api805 sequences, the sequences of the T7 promoter (TAATACGACTCACTATAGGGAGA) and the ribosome binding site (TTTAACTTTAAGAAGGAGATATACAT) in the 5x*OmpA*-Api805 insert could not be altered for each copy (**Supplementary Figure S1**). Thus, there were still homologous sequences in the repeats that could induce deletion of some copies and genetic rearrangement of the insert (Bzymek and Lovett, 2001). However, the incorporation of two copies appears to be genetically stable, which was shown by the isolation of the T7Select-2x*OmpA*-Api805 phage with partially improved activity compared to the T7Select and T7Select-*OmpA*-Api805 phage with only one copy of *OmpA*-Api805. This strategy could be applied to other AMPs, such as CRAMP and melittin, to obtain the desired improvements. Considering the already partially improved activity of the T7Select-(M)CRAMP and T7Select-(M)melittin phages, and the fact that the incorporation of two copies of an AMP into the phage genome appears to be stable and improves the activity, two copies may be sufficient to increase (M)CRAMP and (M)melittin expression and provide more reproducible activity of these phages.

The third approach, the direct attachment of Api805 peptide to the viral capsid via a cleavable linker, did not yield infectious phages. We hypothesize that the attachment of apidaecin reduces the production of the capsid protein below a critical level and that the insolubility, misfolding, or additional high positive charge of the gene10-Api805 construct disrupts the assembly of the T7 capsid shell, preventing the formation of infectious phages. If the high net positive charge of the attached Api805 (+5 under physiological conditions) prevents proper phage formation, this also would most likely apply to other cationic AMPs, including CRAMP and melittin with net charges of +6 and +5, respectively (Ramalingam et al., 1992; Hancock and Diamond, 2000; Islam et al., 2023).

Besides the combinatorial use of bacteriophages and AMPs, the insertion of coding sequences for AMP into the phage genome is an elegant and innovative approach combining these antimicrobial agents. However, further studies are necessary to optimize the reproducibility of the *in vitro* activity of the AMP-phages presented here and to investigate their effectiveness in an infection model.

## Data Availability

The original contributions presented in the study are included in the article/**Supplementary Material**. Further inquiries can be directed to the corresponding author.
