# Supplementary material for: Analysis of engineered T7 bacteriophages containing genetic sequences encoding antimicrobial peptides
Source: Front Antibiot. 2025 Jan 15;3:1515874. doi: 10.3389/frabi.2024.1515874 (PMC11774846; doi:10.3389/frabi.2024.1515874)
Supplement: Supplementary file 1 [file SupplementaryFile1.docx]

Supplementary Material

# Table of Contents

File S1: Material and Chemicals - Reagents 2

Table S1: Bacterial and phage strains, plasmids, and primers 3

Figure S1: Sequence of 5x*OmpA*-Api805 4

Figure S2: MS Analysis of *E. coli* Rosetta pLys lysate and culture supernatant 5

Figure S3: MS Analysis of engineered T7Select phage lysates 7

Figure S4: Bacterial growth of *E. coli* Rosetta cultures infected with T7Select phages 8

Figure S5: DNA sequence alignment of 5x*OmpA*-Api805 and insert 2x*OmpA*-Api805 9

Figure S6: Original SDS-PAGE gel used for Figure 3B 11

Figure S7: Original SDS-PAGE gel used for Figure 3C 12

Figure S8: Original SDS-PAGE gels used for Figure 3D 13

Figure S9: Original agarose gels used for Figure 4B 14

Figure S10: Original agarose gel used for Figure 5A 15

Figure S11: Original agarose gels used for Figure 5B 16

Figure S12: Original agarose gel used for Figure 5C 17

Figure S13: Original agarose gel used for Figure 5D 18

**File S1: Materials and Chemicals**

Reagents were obtained from the following companies unless stated otherwise: AppliChem GmbH (Darmstadt, Germany): Ethidiume Bromide Solution (1%), tris(hydroxymethyl)aminomethane (Tris); Biosolve BV (Valkenswaard, Netherlands): dimethylformamide (DMF, peptide synthesis grade), acetonitrile (HPLC-S gradient grade), formic acid; Bio-Rad Laboratories GmbH (Munich, Germany): Precision Plus Protein™ Dual Xtra protein standard; Carl Roth GmbH, Karlsruhe, Germany): lysozyme (≥45 000 FIP U/mg), Lysogeny broth (LB) Miller, Agar-Agar Kobe I, IPTG (≥99%), kanamycin, phosphate buffered saline (PBS, pH 7.4), potassium chloride (≥99%), potassium dihydrogen phosphate (≥99%), magnesium chloride (≥99%), sodium dodecyl sulfate (SDS, >99.5%), sodium hydroxide (≥98%), trichloroacetic acid (≥99%); Greiner Bio-One GmbH (Frickenhausen, Germany): 96-well microtiter plates; Honeywell Fluka™ (Seelze, Germany): ammonium chloride (≥99.8%), ammoniumbicarbonate, calcium chloride (≥ 99.5 %), magnesium chloride (≥99%); Iris Biotech (Marktredwitz, Germany): leucin-Wang resin; Merck KGAa (Darmstadt, Germany): diethyl ether (puriss); MultiSynTech GmbH: 4-benzyloxybenzyl alcohol (Wang) resin; Orpegen Pharma GmbH (Heidelberg, Germany) or MultiSynTech GmbH (Witten, Germany) or Iris Biotech: all 9-fluorenylmethoxycarbonyl- (Fmoc ) protected amino acids; Phenomenex Inc. (Torrance, CA, USA): Jupiter C18-columns (internal diameter (ID): 21.2 mm, length: 250 mm, particle size: 15 µm, pore size: 30 nm; ID: 10 mm, length: 250 mm, particle size: 5 µm, pore size: 30 nm; ID: 2 mm, length: 150 mm, particle size: 5 µm, pore size: 30 nm); SERVA electrophoresis GmbH (Heidelberg, Germany): acrylamide/bisacrylamide (30% T, 2.67% C), agarose, TEMED, ammonium persulfate (99%), glycine (98.5-101%), Protease inhibitor mix, Tween® 20 (pure), Trypsin (sequencing grade, MS approved), Coomassie Brilliant blue G250; Sigma-Aldrich GmbH (Taufkirchen, Germany): 1,2-ethanedithiole (≥98%), m-cresole (99%), thioanisole (≥99%), N,N-diisopropylcarbodiimide (DIC, >98% by GC), 1-hydroxy-benzotriazole (HOBt, >98%), trifluoroacetic acid (TFA, UV-grade for HPLC), TFA (purum) for peptide synthesis; magnesium sulfate (>97%), potassium chloride (>99%), potassium phosphate (≥ 99 %), sodium acetate (>99%), sodium chloride (≥99,5%), disodium hydrogen phosphate × 12 H2O (≥99%), 2-mercaptoethanol (≥ 99%), Mueller Hinton broth II (MHBII), Tris-acetate EDTA-Buffer (10x, TAE); Thermo Scientific GmbH (Schwerte, Germany): Phusion High-Fidelity DNA Polymerase (2U/µl), dNTP Mix (2 mmol/L each), DNase I (RNase-free, 1 U/μL); VWR (Dresden, Germany): chloroform (≥99%).

**Table S1:** List of all bacterial and phage strains, plasmids, and primers used in the current study.

|  | **Genotype/Sequence** | **Reference** |
| --- | --- | --- |
| **Bacterial Strain** |  |  |
| *E.coli* DH5α | F^–^ *endA1* *glnV44* *thi-1* *recA1* *relA1* *gyrA96* *deoR* *nupG* *purB20* φ80d*lacZ*ΔM15 Δ(*lacZYA-argF*)U169, hsdR17(*r_K_*^–^*m_K_*^+^), λ^–^ | Invitrogen |
| *E.coli* Rosetta^TM^ pLysS | F^–^ *ompT* *gal* *dcm* *hsdS_B_*(*r_B_*^–^*m_B_*^–^) λ(DE3) [*malB*^+^]_K-12_(λ^S^) pLysSRARE(Cm^R^) | Merck |
| **Phage Strain** |  |  |
| T7Select^TM^ 415 | T7 Δgp0.3-07, Δgp3.8, gene10B+MCS | Merck |
| T7Select-*OmpA*-Api805 | + Biotin s1.3 *OmpA*-Api805 | This publication |
| T7Select-2x*OmpA*-Api805 | + Biotin s1.3 2x*OmpA*-Api805 |  |
| T7Select-(M)CRAMP | + Biotin s1.3 (M)CRAMP |  |
| T7Select-(M)melittin | + Biotin s1.3 (M)melittin |  |
| **Plasmids** |  |  |
| pApi801(G1M) / pApi802 | pET28a^+^ Biotin s1.3 Api802 Kan^R^ | Ludwig et al., 2022b |
| pApi805(G1M) / pApi806 | pET28a^+^ Biotin s1.3 Api806 Kan^R^ |  |
| p*OmpA*-Api805 | pET28a^+^ Biotin s1.3 *OmpA*-Api805 Kan^R^ | This publication |
| p5x*OmpA*-Api805 | pET28a^+^ Biotin s1.3 5x*OmpA*-Api805 Kan^R^ |  |
| pgene10 | pET28a^+^ His_6_-gene10 Kan^R^ |  |
| pgene10-Api805 | pET28a^+^ His_6_-gene10-Api805 Kan^R^ |  |
| p(M)CRAMP | pET28a^+^ Biotin s1.3 (M)CRAMP Kan^R^ |  |
| p(M)melittin | pET28a^+^ Biotin s1.3 (M)melittin Kan^R^ |  |
| **Primer** |  |  |
| pET28 fwd | ttatgctagttattgctcagcgg | Ludwig et al., 2022b |
| pET28 rev | CggTGATGTCGGCGATATAGG |  |
| T7 fwd | ggagctgtcgtattccagtc |  |
| T7 rev | aacccctcaagacccgttta |  |

References

Ludwig, T., Hoffmann, R., and Krizsan, A. (2022b). Construction and Characterization of T7 Bacteriophages Harboring Apidaecin-Derived Sequences. Current issues in molecular biology, 44(6), 2554–2568. https://doi.org/10.3390/cimb44060174

**Figure S1. A** Schematic illustration of the DNA fragment of the 5x*OmpA*-Api805 insert, which encodes five copies of *OmpA*-Api805 by five different DNA sequences using different codons to avoid multiple replication. **B** DNA sequence of 5x*OmpA*-Api805.

**
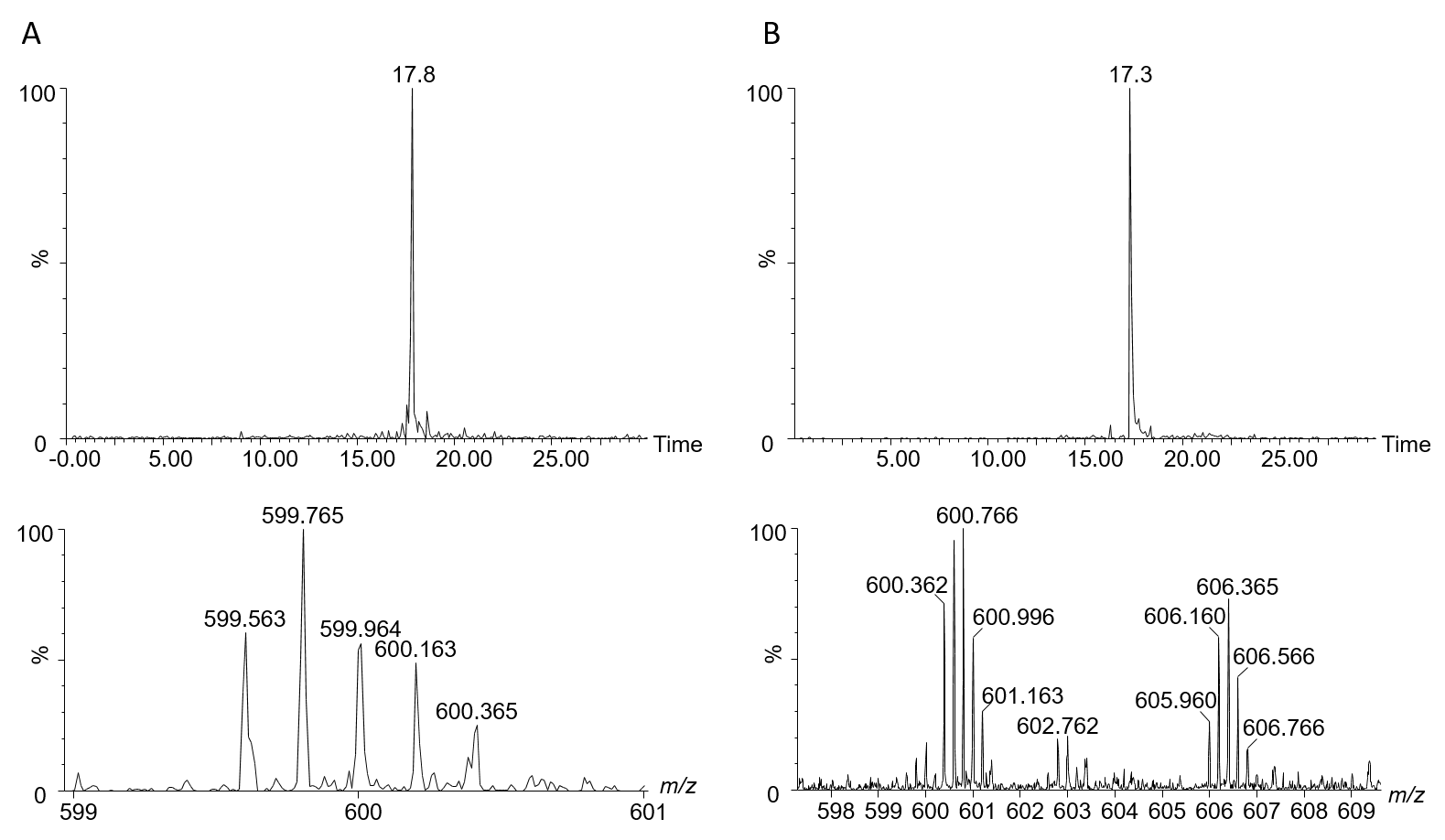
**

**
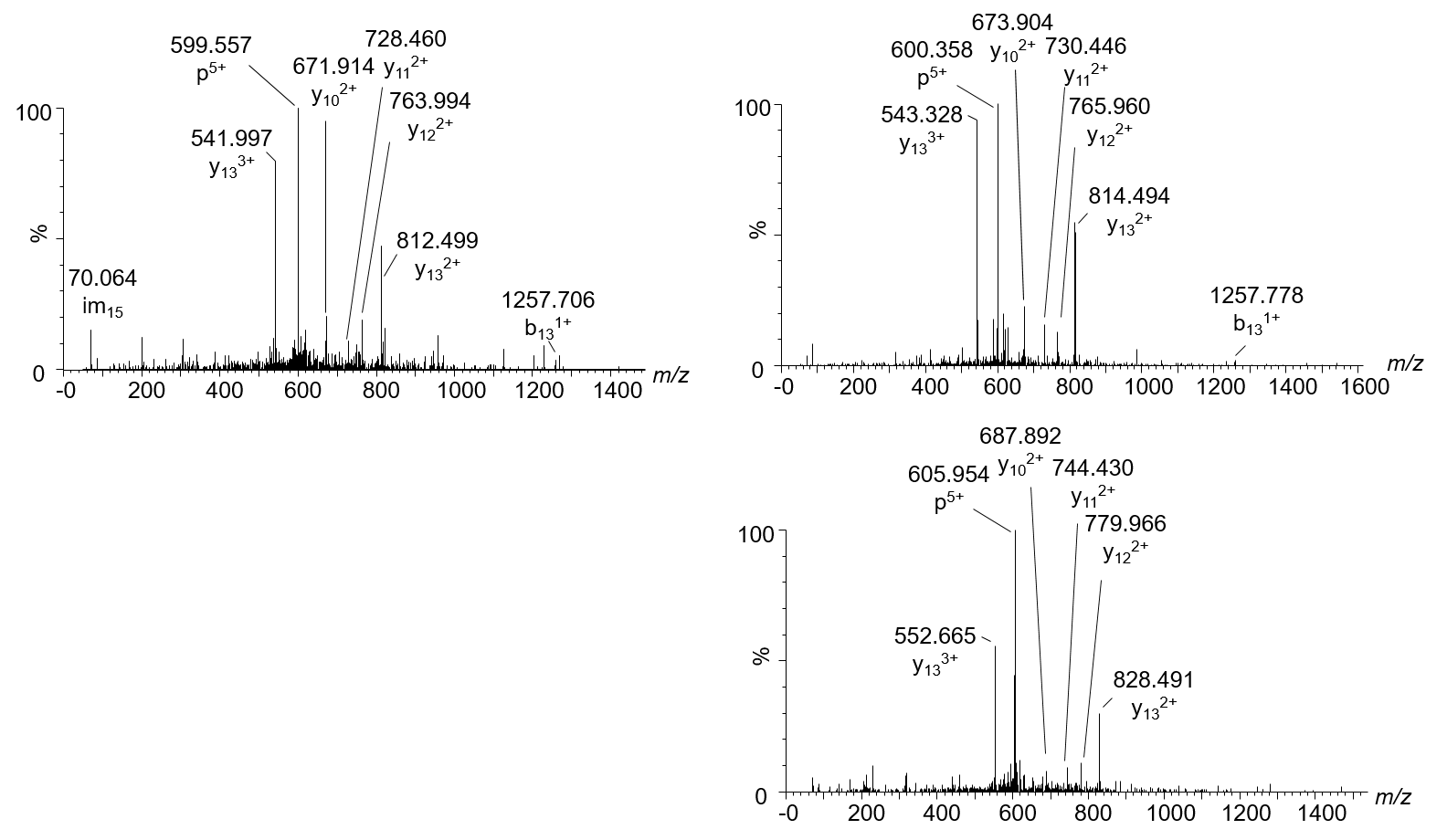
**

**Figure S2. A** Mass spectrometry analysis of the lysate of *E. coli* Rosetta pLys expressing (M)melittin after lysing the residual cell pellet. XIC (top) of *m/*z 599.563 (middle) which fits to the five times charged ion with methionine oxidized (theor. [M+H]^5+^ 596.363; with methionine oxidation (position 1 Met +15.999; [M+H]^5+^599.562), which was confirmed by MS/MS (bottom) where b- and y-ion series confirmed the modification. As methionine can be modified by oxidation quite easily it could be an artifact of sample clean up and handling. **B** Mass spectrometry analysis of the culture supernatant of *E. coli* Rosetta pLys expressing (M)melittin. Compared to the bacterial lysate (m)mellitin was found even more modified (middle). Additional to methionine oxidation, tryptophan in position 20 was modified either to kynurein (+ 3.989) or N-formylkynurein (+ 31.999), which results in [M+H]^5+^ 600.362 (theor. 600.361) or 605.96 (theor. 605.96). These modified versions co-eluted 0.5 minutes earlier than peptide just oxidized at methionine (top) due the greater hydrophilicity of the peptide. Both modification variants could be confirmed by MS/MS as several y-ions confirmed the mass shift corresponding to kynurein (bottom upper MS/MS spectra) or N-formylkynurein (bottom lower MS/MS spectra).

**
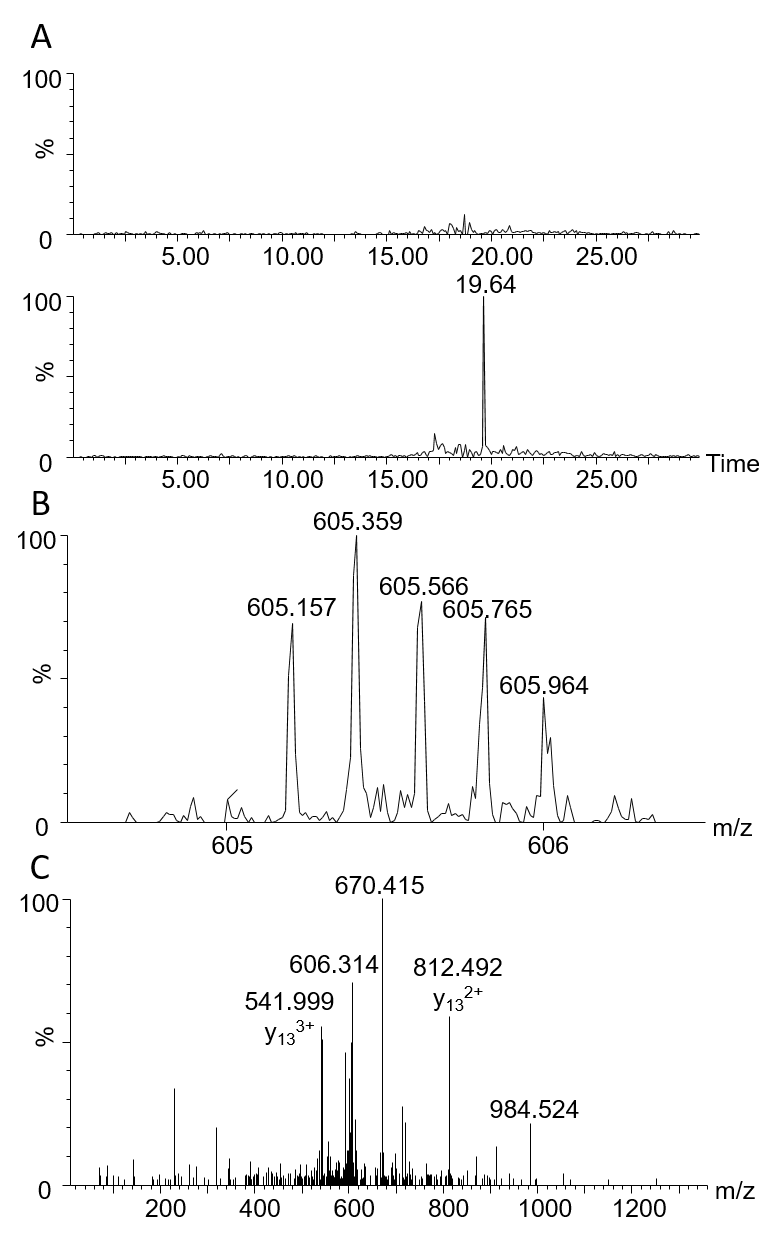
**

**Figure S3.** Mass spectrometry analysis of engineered T7Select phage lysates. **A** XIC for *m/z* 605.157 from T7Select phage lysat without insert (upper panel) and T7Select-(M)melittin phage lysat. **B** Isotop pattern of *m/z* 605.157 which fit to the value of [M+H]^5+^ of (M)melittin carboxylated on methionine (+ 44.990) 605.161. **C** MS/MS spectra of *m/z* 605.157 indicating with y_13_^3+^ and y_13_^2+^ the presence of (M)melittin sequence from position 15-27 (PALISWIKRKRQQ). However, it does not proof the mass shift of + 43.990 to be a carboxylation of methionine N-term.

**Figure S4.** Bacterial growth of *E. coli* Rosetta cultures infected with engineered T7Select phages (MOI 0.0625) without insert (gray) and with inserts (red) encoding *OmpA*-Api805, (M)CRAMP or (M)melittin. Shown are OD_600_ values recorded every 15 min for 36 h. Experiments were performed three times in triplicate. Shown are the mean curves of all nine replicates with standard deviation (SD) and the curves of the individual replicates of each experiment.

**Figure S5.** Sequence alignment of the DNA fragment of the 5x*OmpA*-Api805 insert and the insert integrated into the T7Select-2x*OmpA*-Api805 genome. The restriction sites of Sall (GTCGAC) and HindIII (AAGCTT) are shown in dark blue. Codon adjusted intermediate sequences are shown in black. Sequences for the T7 promoter (TAATACGACTCACTATAGGGAGA) and the ribosome binding site (TTTAACTTTAAGAAGGAGATATACAT) are shown in gray. Codon adjusted *OmpA*-Api805 copies are shown in red (copy one), green (copy two), blue (copy three), purple (copy four), and orange (copy five).

**Figure S6.** Original SDS-PAGE gel used for Figure 3B. Lane 1 (Protein Ladder), lane 4 (Rosetta pLys pET28a(+)-gene10-native, -IPTG), lane 5 (Rosetta pLys pET28a(+)-gene10-native, +IPTG), lane 8 (Rosetta pLys pET28a(+)-gene10-Api805, -IPTG), and lane 9 (Rosetta pLys pET28a(+)-gene10-Api805, +IPTG) were excised, merged and the areas corresponding to the proteins were cropped. Lanes 2, 3, 6, and 7 were not used and are not relevant to the manuscript.

**Figure S7.** Original SDS-PAGE gel used for Figure 3C. Lane 1 (Protein Ladder), lane 6 (Rosetta pLys pET28a(+)-gene10-native, +IPTG, soluble fraction), lane 7 (Rosetta pLys pET28a(+)-gene10-Api805, +IPTG, soluble fraction), lane 10 (Rosetta pLys pET28a(+)-gene10-native, +IPTG, insoluble fraction), and lane 11 (Rosetta pLys pET28a(+)-gene10-Api805, +IPTG, insoluble fraction) were excised, merged and the areas corresponding to the proteins were cropped. Lanes 2, 3, 4, 9, and 12 were not used and are not relevant to the manuscript.

**Figure S8.** Original SDS-PAGE gels used for Figure 3D. Lane 1 (Protein Ladder), lane 2 (Rosetta pLys pET28a(+)-*OmpA*-Api805, -IPTG, load: 10 µL), lane 3 (Rosetta pLys pET28a(+)-5x*OmpA*-Api805, -IPTG, load: 10 µL), lane 4 (Rosetta pLys pET28a(+)-(M)CRAMP, -IPTG, load: 10 µL), lane 5 (Rosetta pLys pET28a(+)-*OmpA*-Api805, +IPTG, load: 10 µL), lane 6 (Rosetta pLys pET28a(+)-(M)melittin, -IPTG, load: 10 µL), lane 7 (Rosetta pLys pET28a(+)-5x*OmpA*-Api805, +IPTG, load: 10 µL), lane 10 (Rosetta pLys pET28a(+)-(M)CRAMP, +IPTG, load: 10 µL), lane 11 (Rosetta pLys pET28a(+)-(M)melittin, +IPTG, load: 10 µL), lane 18 (Protein Ladder), lane 20 (1 µg Api805), lane 21 (1 µg CRAMP), and lane 22 (1 µg melittin) were excised, merged and the corresponding areas were cropped. Lanes 8, 9, 12-17, and 19 were not used and are not relevant to the manuscript. Lane 14 (Rosetta pLys pET28a(+)-(M)CRAMP, +IPTG, load: 20 µL), lane 15 (Rosetta pLys pET28a(+)-*OmpA*-Api805, +IPTG, load: 15 µL), Lane 16 (Rosetta pLys pET28a(+)-(M)melittin, +IPTG, load: 15 µL), and lane 17 (Rosetta pLys pET28a(+)-(M)melittin, +IPTG, load: 5 µL) were the same samples loaded on both gels but in different amounts.

**Figure S9.** Original agarose gels used for Figure 4B. Lane 1 (DNA Ladder 100 bp), lane 2 (T7Select, load: 10 µL), lane 11 (T7Select-*OmpA*-Api805, load: 10 µL), lane 12 (T7Select-(M)melittin, load: 10 µL), and lane 14 (T7Select-(M)CRAMP, load: 10 µL) were excised, merged and the corresponding areas were cropped. Lane 3 (T7Select-(M)melittin, load: 10 µL), lane 4 (T7Select-(M)melittin, load: 10 µL), lane 5 (T7Select, load: 5 µL), lane 6 (T7Select-(M)melittin, load: 5 µL), lane 7 (T7Select-(M)melittin, load: 5 µL), lane 8 (DNA Ladder 100 bp), lane 9 (T7Select-*OmpA*-Api805, load: 10 µL), lane 10 (T7Select-*OmpA*-Api805, load: 10 µL), lane 13 (T7Select-(M)melittin, load: 10 µL), lane 15 (T7Select-(M)CRAMP, load: 10 µL), and lane 16 (T7Select, load: 10 µL) were not used.

**Figure S10.** Original agarose gels used for Figure 5A. Lane 10 (pET28(+)-5x*OmpA*-Api805, load: 10 µL), lane 11 (DNA Ladder 100 bp), and lanes 13-25 (T7Select-5x*OmpA*-Api805, Lysates 1-13 isolated from plaques, load: 10 µL), were excised, merged and the corresponding areas were cropped. Lane 1 (DNA Ladder 1000 bp), lanes 2-7, lane 8 (pET28(+)-*OmpA*-Api805, load: 10 µL), lane 9 (pET28(+)-5x*OmpA*-Api805, load: 5 µL), lane 12 (DNA Ladder 1000 bp), and lane 26 (DNA Ladder 100 bp) were not used.

**Figure S11.** Original agarose gels used for Figure 5B. Lanes 4-14 (T7Select-5x*OmpA*-Api805 [plaque 10], Lysates 1-11 isolated from plaques, load: 10 µL), and lanes 17-30 (T7Select-5x*OmpA*-Api805 [plaque 10], Lysates 12-25 isolated from plaques, load: 10 µL) were excised, merged and the corresponding areas were cropped. Lane 1 (DNA Ladder 1000 bp), lane 2, lane 3, lane 15 (DNA Ladder 100 bp), and lane 16 (DNA Ladder 1000 bp) were not used.

**Figure S12.** Original agarose gel used for Figure 5C. Lane 1 (DNA Ladder 1000 bp), lane 3 (T7Select), lane 4 (T7Select-2x*OmpA*-Api805, lysate isolated from plaque 23) and lane 9 (DNA Ladder 100 bp) were excised, merged and the corresponding areas were cropped. Lanes 2, 5, 8 (all no load), lane 6 (T7Select), and lane 7 (T7Select-2x*OmpA*-Api805, lysate isolated from plaque 23) were not used.

**Figure S13.** Original agarose gel used for Figure 5D. Lane 1 (DNA Ladder 1000 bp), lane 3 (T7Select-*OmpA*-Api805), lanes 4-11 (T7Select-2x*OmpA*-Api805, lysates 1-8 isolated from plaques), lanes 14-16 (T7Select-2x*OmpA*-Api805, lysates 9-11 isolated from plaques), lanes 18-21 (T7Select-2x*OmpA*-Api805, lysates 12-15 isolated from plaques) and lane 22 (DNA Ladder 100 bp) were excised merged and the corresponding areas were cropped. Lane 2 (T7Select), lane 11 (DNA Ladder 100 bp), lane 12 (T7Select), lane 13 (T7Select-*OmpA*-Api805) and lane 17 (T7Select-5x*OmpA*-Api805, lysate from plaque 10, was load as a reference with one, two and four copies of *OmpA*-Api805) were not used.
